# Exploring the Impact of Prior Beta-Blocker and Calcium Channel Blocker Usage on Clinical Outcomes in Critically Ill Patients With Sepsis: An Observational Study

**DOI:** 10.7759/cureus.46169

**Published:** 2023-09-28

**Authors:** Sachin Kumar, Deepak Malviya, Manoj Tripathi, Sujeet Rai, Soumya S Nath, Shiv Shanker Tripathi, Smarika Mishra

**Affiliations:** 1 Department of Anaesthesiology, Rama Medical College Hospital and Research Centre, Kanpur, Kanpur, IND; 2 Department of Anaesthesia and Critical Care, Dr. Ram Manohar Lohia Institute of Medical Sciences, Lucknow, IND; 3 Department of Emergency Medicine, Dr. Ram Manohar Lohia Institute of Medical Sciences, Lucknow, IND

**Keywords:** beta-blockers, calcium channel blockers, sepsis, hospital mortality, intensive care units

## Abstract

Background

Sepsis is associated with increased Ca^++^ levels in many cell types that can cause cytotoxicity and cell death through multiple mechanisms. In patients with sepsis, limiting beta-adrenergic stimulation may also be beneficial. The intense adrenergic stimulation of sepsis results in cardiac and extra-cardiac effects. In the intensive care unit (ICU), the question of whether to continue calcium channel blockers (CCBs) and beta-blockers in patients with sepsis who were using these medications before ICU admission is of significant concern.

Methodology

In this prospective observational study, we have included 114 patients who met the inclusion criteria of being diagnosed as having sepsis, aged 18 to 65 years, and expected to stay in the ICU for more than 72 hours. These patients were divided into three groups: group 1 consisted of patients taking CCBs before admission, group 2 included those taking beta-blockers before admission, and group 3 served as the control group, comprising patients who had not taken either of these medications before admission. Disease severity in the ICU was assessed and documented by the Sequential Organ Failure Assessment (SOFA) score. Clinical outcomes among three groups were compared regarding the need for vasopressor support, serum procalcitonin (PCT), serum lactate, serum quantitative C-reactive protein (qCRP), SOFA score, and 28 days mortality. Parametric data were expressed as mean ± standard deviation. The Kruskal-Wallis test was used to analyze parametric data between the two groups and among three groups.

Results

Mortality was found lower in group 1 (21.05%) and group 2 (26.31%) than in group 3 (47.36%), and this association was found to be statistically significant (*P *= 0.033). We also found a significant difference in mortality between groups 1 and 3 (*P *= 0.015) and no significant difference between groups 2 and 3 (*P *= 0.057). Mortality was found to be significantly associated with high SOFA scores on days 1, 3, and 7.

Conclusions

From the aforementioned results, we concluded that the mortality rate in patients with sepsis was improved when they were pretreated with beta-blockers or CCBs before admission to the ICU and that medication should be continued if not contraindicated in the ICU course.

## Introduction

Sepsis is a substantial health burden worldwide. More than 19 million sepsis (formerly severe sepsis) cases and 5 million sepsis-related deaths are estimated to occur annually, the majority in low and middle-income countries [1]. In the United States, sepsis is the most common cause of in-hospital deaths and costs more than US$24 billion annually [2]. Infection-prevention efforts, including those targeting both community-acquired and healthcare-associated infections, can reduce sepsis incidence [3,4]. Sepsis is treatable, and timely implementation of targeted interventions improves outcomes. Patients with sepsis can go into septic shock, acute respiratory distress syndrome, and multi-organ failure, ultimately leading to death.

Sepsis is defined as a life-threatening condition characterized by organ dysfunction resulting from a dysregulated host response to infection. Organ dysfunction can be identified as an acute increase in the Sequential Organ Failure Assessment (SOFA) score by 2 points or more, and it is associated with an in-hospital mortality rate exceeding 10%. Septic shock should be defined as a subset of sepsis in which particularly profound circulatory, cellular, and metabolic abnormalities are associated with a greater risk of mortality than with sepsis alone [5]. Sepsis is associated with increased Ca^++^ levels in many cell types that can cause cytotoxicity and cell death through multiple mechanisms, including mitochondrial dysfunction, nuclear injury, cytoskeleton disruption, increased nitric oxide and pro-inflammatory cytokine production, and enhanced apoptosis [6,7,8]. Therefore, calcium channel blockers (CCBs)can restore such disrupted cellular processes to their normal states through calcium channel-dependent calcium ion homeostasis. Furthermore, CCBs exert pleiotropic effects, such as antioxidant effects [9] and immunodepression and anti-inflammatory activity suppression [10], in sepsis. Therefore, CCB use may benefit patients with sepsis.

In patients with sepsis, limiting beta-adrenergic stimulation may also be beneficial [11]. The intense adrenergic stimulation of sepsis results in cardiac (increased contractility, heart rate, and myocardial energy demand) and extracardiac (catabolic state, hyperglycemia, hypercoagulability, and release modulation of systemic inflammatory cytokines) effects [12,13]. When sepsis progresses, or tachycardia persists after fluid resuscitation and pain/agitation control, cardiac energy demand can overcome supply with the risk of cardiac dysfunction and multiorgan failure [14]. The physiologic rationale behind the clinical application of beta-blockers in septic shock is the modulation of the cardiac and extracardiac effects. It has been suggested that beta-blockers can counteract the hypermetabolism of the hyperdynamic phase of sepsis to prevent the catabolic phase of the decompensated period of sepsis [15]. Most of the previous studies have compared either beta-blockers or CCBs with control for assessment of mortality. In this study, we compared beta-blockers and CCBs with control and between beta-blockers and CCBs.

## Materials and methods

This monocentric comparative prospective observational study was conducted in the Department of Anaesthesiology and Critical Care Medicine in a tertiary care hospital and research center in North India. After approval from our institutional ethical committee (IEC No. 74/17), the study was done over one year, starting from August 2019 to July 2020. This study was enrolled in a clinical trial registry (CTRI/2019/08/021007), and we have strictly followed the ethical principles for medical research involving human subjects according to the Helsinki Declaration 2013.

After obtaining consent from the patient’s relative/guardian, we included adult patients (aged 18-65 years) admitted to our ICU with a primary or concurrent clinical diagnosis of sepsis, as defined by the Surviving Sepsis Campaign 2016 (Sepsis-3) [5], who were expected to stay in the ICU for ≥72 hours and had been taking CCBs or beta-blockers before ICU admission. We excluded patients from the study if they had negative consent from their relatives, required inotropic support (noradrenaline) for hypotension at a dose of ≥0.1 mcg/kg/minute during their ICU stay [16], had septic shock, were previously diagnosed with sepsis at another center, or if beta-blockers or CCBs were used during their ICU stay or thereafter.

**Figure 1:**
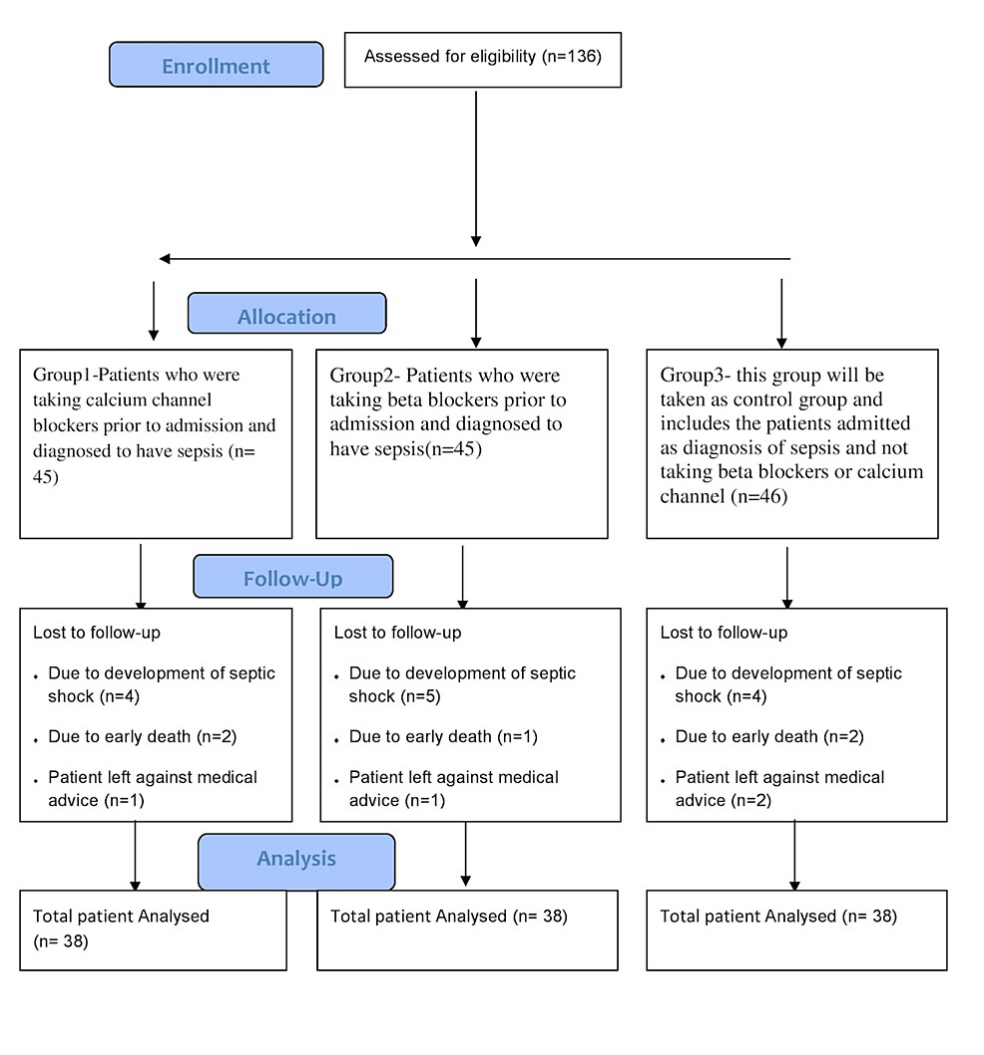
Consort diagram showing the number of patients included and finally analyzed. Image credit: Manoj Tripathi.

As shown in the consort diagram (Figure 1), 136 patients enrolled in the study with sepsis as a primary or concurrent diagnosis. Sepsis was diagnosed according to the definition of Surviving Sepsis Campaign 2016 (Sepsis-3) [5]. After taking proper history, clinical examination, routine laboratory investigations, and arterial blood gas (ABG) analysis, including serum lactate, disease severity was assessed with the help of the SOFA score. Among recruited patients, five expired within 24 hours, four were discharged as left against medical advice (LAMA) on the second day of ICU admission, and 13 patients developed septic shock within 72 hours. Thus, a total of 114 patients were included in this study who met all inclusion criteria. Patients were divided into three groups, each consisting of 38 individuals, based on their prior use of beta-blockers, CCBs, or neither of these medications, as follows:

· Group 1: This group consisted of patients who were taking CCBs before admission and diagnosed with sepsis.

· Group 2: This group consisted of patients who were taking beta-blockers before admission and diagnosed with sepsis.

· Group 3: This group was taken as a control group and included the patients admitted with a diagnosis of sepsis and not taking beta-blockers or CCBs.

After admission to the ICU of our institution, all the critically ill patients were evaluated with a thorough physical examination and laboratory investigations such as complete blood count, renal function test, liver function test, serum electrolytes, blood culture, and sensitivity for aerobic and anaerobic bacteria from two sites, and chest X-ray was done in all patients. ABG analysis was done at admission and followed up in the morning and evening during the ICU stay. At the time of admission, one researcher assessed the severity of illness by SOFA score and Acute Physiology and Chronic Health Evaluation II (APACHE II) score. Blood samples for biomarkers like procalcitonin (PCT), serum lactate, and quantitative C-reactive protein (qCRP) measurements were taken at the time of admission to the ICU before the administration of the first dose of antibiotics. These parameters were repeated at the time of admission (D1), 72 hours later (D3), and again on day 7 (D7) of ICU treatment. The need for vasopressor, that is, noradrenaline infusion (<0.1 mcg/kg/minute) was also compared among the groups. GCS scoring, PaO2/FiO2 ratio assessment, hemodynamic monitoring, fluid intake and urine output monitoring, invasive arterial blood pressure monitoring, and CVP monitoring were also conducted. SOFA score was determined on D1, D3, and D7 and during the patient's follow-up until their outcome. Twenty-eight days of mortality was assessed by analyzing the obtained data. The study's primary objective was to observe 28 days of mortality among three groups. The study's secondary objective was to assess different biomarkers like PCT, qCRP, lactate, and SOFA score with the association of SOFA score with mortality.

A previous study showed a 28-day mortality rate of approximately 50% (0.5) in patients with sepsis receiving esmolol [17]. Another study by Wiewel et al. showed 30 30-day mortality rate for patients with sepsis with the use of CCBs 20% (0.2) [16]. If the actual difference between the mortality rate for both these groups is 30% (0.3), we need to study 38 patients in each group to be able to reject the null hypothesis that the mortality rate for the patients in these groups is equal to a probability (power) of 80% (0.8). The type 1 error probability associated with this null hypothesis test is 0.05. 

Targeting a sensitivity of 80% of novel parameters with an observed sepsis prevalence of 28% in ICU-admitted adult patients, the sample size was calculated using the following formula:

*n* = (*Zα*/2+ *Zβ*)2 × P1 (1-P1) + P2 (1-P2) × (*r* + 1)/*r* × (P1-P2)2

where *Z *is the 95% confidence interval.

After calculation, we found *n* = 37.8 (rounding up to 38).

Parametric data were expressed as mean ± standard deviation. The Kruskal-Wallis test was used to analyze parametric data between the two groups and among three groups. The chi-square test was used for the analysis of continuous and categorical variables. A *P*-value of <0.05 was considered statistically significant, and a *P*-value of >0.05 was not considered statistically significant.

## Results

A total of 136 patients were enrolled in the study. Of the 136 patients, 114 (83.8%) patients were included in the final analysis. In our study, we did not find any statistically significant difference in demographic variables like age, sex, and baseline APACHE II score of the patients(*P *> 0.05). To assess the baseline characteristics of patients, we assessed the APACHE II score. Table 1 shows the comparison of the need for vasopressor less than or equal to 0.1 mcg/kg/minute among the groups and found them to be comparable. The difference was statistically insignificant (*P *= 0.864) on D1, D3, and D7. We also could not find any significant difference between any two groups on D1, D3, and D7. As in Table 2, we compared serum PCT levels among three groups on D1, D3, and D7 and found them statistically insignificant in all periods (*P* > 0.05). But we found a statistically significant difference between groups 1 and 3 (*P *= 0.0323) and between groups 2 and 3 (*P *= 0.0116) at D7. Table 2 also shows the comparison of serum lactate levels among the groups across the periods. No significant difference was found in lactate among the groups (*P *> 0.05) at different periods and between any two groups. A comparison of qCRP among the groups across the periods was also analyzed, as shown in Table 2. No significant (*P *> 0.05) difference was found in qCRP among the groups on D1, D3, and D7. We found a significant difference between groups 1 and 3 (*P *= 0.031) on D1. Table 3 shows the comparison of SOFA scores among the groups across the periods. There was no significant difference (*P* > 0.05) in SOFA scores among the groups at all the periods. As shown in Table 4, we compared SOFA scores between survivors and nonsurvivors in all groups and found statistically significant differences (*P *< 0.001). Table 5 shows the comparison of mortality among the groups. The mortality was lower in group 1 (21.05) and group 2 (26.31%) than in group 3 (47.36%), and this association was found to be statistically significant (*P *= 0.033). We also found a significant difference in mortality between groups 1 and 3 (*P *= 0.015), and no significant difference was found between groups 2 and 3 (*P *= 0.057).

**Table 1:** Comparison of need for vasopressor (noradrenaline) less than 0.1 mcg/kg/minute among the groups. ^*^Chi-square test. No significant difference *P *> 0.05.

Need of vasopressor	Group 1 (*n *= 38)		Group 2 (*n *= 38)		Group 3 (*n *= 38)		*P*-value^*^
	n	%	n	%	n	%	
Yes	9	23.6	8	21.1	10	26.3	0.864
No	29	76.3	30	78.9	28	73.6	

**Table 2:** Comparison of serum PCT (ng/mL), serum lactate (mg/dL), and qCRP (mg/dL) among the groups across the time period. ^*^Kruskal-Wallis test. No significant difference *P *> 0.05. SD, standard deviation; PCT, procalcitonin; qCRP, quantitative C-reactive protein

Serum PCT (ng/mL)	Group 1 (mean ± SD)	Group 2 (mean ± SD)	Group 3 (mean ± SD)	*P*-value^*^
Day 1	25.88 ± 45.29	22.70 ± 29.96	28.24 ± 12.07	0.753
Day 3	15.19 ± 23.52	18.16 ± 26.51	18.71 ± 10.61	0.742
Day 7	8.67 ± 16.70	8.38 ± 14.38	15.02 ± 6.56	0.520
Serum lactate (mg/dL)				
Day 1	25.05 ± 25.37	24.79 ± 19.47	27.11 ± 14.91	0.863
Day 3	14.32 ± 17.71	17.62 ± 16.43	15.05 ± 12.68	0.632
Day 7	9.23 ± 13.62	9.39 ± 9.79	10.11 ± 7.78	0.929
qCRP (mg/dL)				
Day 1	53.73 ± 32.43	59.55 ± 34.53	66.93 ± 17.80	0.147
Day 3	38.88 ± 25.61	45.71 ± 27.97	47.93 ± 14.16	0.217
Day 7	26.52 ± 19.51	29.68 ± 24.44	35.16 ± 11.56	0.145

**Table 3:** Comparison of SOFA scores among the groups across the time period. ^*^Kruskal-Wallis test. No significant difference *P* > 0.05. SD, standard deviation; SOFA, Sequential Organ Failure Assessment

Time period	Group 1 (mean ± SD)	Group 2 (mean ± SD)	Group 3 (mean ± SD)	*P*-value^*^
Day 1	7.34 ± 3.41	7.29 ± 2.98	7.37 ± 3.27	0.994
Day 3	5.69 ± 4.04	6.41 ± 4.52	6.32 ± 4.44	0.734
Day 7	4.78 ± 4.20	4.59 ± 5.11	5.00 ± 5.01	0.933

**Table 4:** Comparisons of SOFA scores between survivors and non-survivors on day 1, day 3, and day 7 in all patients. ^*^Kruskal-Wallis test. Significant difference *P *< 0.05. SD, standard deviation; SOFA, Sequential Organ Failure Assessment

	Survival (*n* = 71)	Nonsurvival (*n* = 43)	*P*-value
	Mean ± SD	Mean ± SD	
SOFA (day 1)	5.2394 ± 1.63414	10.7674 ±1.93756	<0.001^*^
SOFA (day 3)	3.2817 ± 1.62290	11.5263 ± 1.95541	<0.001^*^
SOFA (day 7)	2.4262 ± 1.63767	12.3913 ± 1.46905	<0.001^*^

**Table 5:** Comparison of mortality among the groups. ^*^Chi-square test.  Significant difference *P *< 0.05.

Mortality	Group 1 (*n *= 38)		Group 2 (*n *= 38)		Group 3 (*n *= 38)		*P*-value*
	n	%	n	%	n	%	
Expired	8	21.05	10	26.31	18	47.36	0.033
Discharged	30	78.95	28	73.69	20	52.64	

## Discussion

In this study, we compared the effect of continuation of preadmission beta-blockers and CCBs on patients with sepsis on mortality, biomarkers, and SOFA scores. The study's primary endpoint was a comparison of mortality among the groups. The mortality was found to be lower in group 1 (21.05%) and group 2 (26.31%) than in group 3 (47.36%). Maximum mortality was seen in group 3, and minimum mortality was seen in group 1, and this association was found significant (*P *= 0.033). In an observational study involving critically ill patients with sepsis admitted to the ICU with prior use of CCBs, improved 30-day survival was observed in the CCB group [16]. Similarly, in another study, it was concluded that preadmission CCB use was associated with a lower risk of contracting bacteremia, a lower risk of acute respiratory insufficiency, and a lower risk of ICU admission [18]. A study showed that patients previously prescribed beta-blockers had lower mortality at 28 days than those previously untreated and found a 28-day survival advantage in patients who were taking beta-blockers at the time of admission and who subsequently developed sepsis. Our results also found decreased mortality in the beta-blocker-taking group than in the control group, but it was not statistically significant [19].

The need for vasopressor support during ICU stay among the groups was compared, and we found that 23.6% of group 1, 21.1% of group 2, and 26.3 % of group 3 needed vasopressor support. However, the difference was statistically insignificant (*P *> 0.05). We did not find any studies supporting the use of CCBs to reduce the need for vasopressors during the ICU stay of patients with sepsis. One study found less vasopressor requirement in beta-blocker-treated patients, which was statistically insignificant (*P *> 0.05). The results were similar and supported the results of our study [19]. The mechanism of this could decrease cellular oxygen expenditure. Improvement in microvascular flow was reported in one study [17], which could imply an improvement in cellular oxygen delivery.

It was seen that the serum PCT level was high in all three groups, but a lesser value of serum PCT was seen in group 2 and group 1 than in group 3 across the time period and was found statistically insignificant. Our results are comparable to the results of a prospective observational study of 296 patients with severe sepsis or septic shock with preexisting, chronic oral beta-blocker therapy. Continuation of beta-blocker therapy was significantly associated with a decreased hospital stay and 28-day and 90-day mortality rates in contrast to beta-blocker cessation [20]. One researcher reported significantly less rise in serum PCT levels in patients where preadmission CCB was used than in those patients where CCB was discontinued (*P *< 0.05). These results were comparable to our study [16].

In this study, comparison clearly showed that the serum lactate level was high in all three groups, but the extent of the rise was less in group 2 and group 1 than in group 3 across the periods. Our results were comparable to the results of a study [21] in which the authors conducted a prospective observational study on 228 patients to assess the effect of beta-blockers on blood lactate levels and mortality in patients with sepsis. The authors found that change in the blood lactate level was insignificant between beta-blockers and non-beta-blockers groups. In another retrospective cohort study of 260 patients with severe sepsis or septic shock, 25% were previously treated with beta-blockers. They concluded that blood lactate concentration was significantly lower in patients previously treated with beta-blockers [22]. We also found the same results but not statistically significant. This may be attributed to the smaller sample size in our study, as we did not include patients with septic shock or severe sepsis. So, the study population was also different. We could not find any study comparing lactate levels in the preadmission CCB and control groups.

It was seen that qCRP levels were high in all three groups, but a lesser value of qCRP was seen in group 1 (patients on CCB therapy) and group 2 (patients on beta-blocker therapy) than in group 3 across the period. The researcher conducted a retrospective propensity-matched cohort study after screening 2,214 patients hospitalized for pneumonia. Preadmission CCB use was associated with a lower risk of contracting bacteremia, and this was associated with a decreased level of qCRP in a patient in the CCB group than in the non-CCB group, but this difference was insignificant (*P*-value > 0.05) [18]. Our study found a significant difference between the CCB group and the control group on D1 (*P *= 0.031). This difference may be due to our study's reduced sample size and different groups of patients.

A comparison of SOFA scores was also made among the groups across the periods. There was no significant (*P *> 0.05) difference in SOFA scores among the groups at all the periods. On D1, the maximum change in SOFA scores was seen in group 3 (7.37 ± 3.27), and the minimum elevation was seen in group 2 (7.29 ± 2.98). Our study shows that the change in SOFA scores was high in all three groups, but a lesser change in SOFA scores was seen in group 2 and group 1 than in group 3 across the period. We also compared SOFA scores between survivors and nonsurvivors. We found a very high SOFA score in nonsurvivors than in survivors on D1, D3, and D7, which was statistically highly significant (*P *< 0.001). So mortality was found to be positively correlated with a high SOFA score. Our results were comparable to those of Wiewel et al., who concluded improved 30-day survival in patients with prior use of CCBs. They found that there was no significant difference in SOFA scores between patients who had been taking CCBs before admission and those who had not (*P*-value = 0.67) [16].

The multicentric trial was needed to confirm the findings and more variables could have been taken into account, like the duration of ICU stay and the duration of days on mechanical ventilation. We did not take into account the specified beta-blockers or CCBs and their impact on clinical outcomes. We also did not consider the duration of beta-blocker and CCB therapy. These are the limitations of our study.

## Conclusions

We conclude from our study that a significantly reduced mortality rate was found in patients with sepsis who were pretreated with beta-blockers or CCBs. Mortality was found to be reduced in patients being treated with CCBs than beta-blockers. Clinical parameters were found better in patients pretreated with CCBs or beta-blockers than in control group patients.
